# Supplementary material for: Survival benefit from adjuvant chemoradiotherapy in local advanced gastric cancer without accurate D2 confirmation: a real-world retrospective study (TJ-ARK01)
Source: PeerJ. 2025 Jul 10;13:e19363. doi: 10.7717/peerj.19363 (PMC12256042; doi:10.7717/peerj.19363)
Supplement: Supplemental Information 1 — Table S1. Toxicity Profile (all grades). Table S2. Univariate and Multivariate analyses of prognostic factors for all patients for DFS. Table S3. Univariate and Multivariate analyses of prognostic factors for all patients for OS. TableS4. Pattern of Recurrence in all patients. Table S5. Pattern of Recurrence in patients with different staging. Table S6. Pattern of Recurrence in patients with different staging. Table S7. Baseline of lymph node invasion between TJ-ARK01, ARTIST1 and ARTIST2. [file peerj-13-19363-s001.docx]

Supplementary material

Table S1. Toxicity Profile (all grades)

|  | CT Arm(n=162) | | | | | | | | CRT Arm(n=166) | | | | | | | |
| --- | --- | --- | --- | --- | --- | --- | --- | --- | --- | --- | --- | --- | --- | --- | --- | --- |
|  | Grade1 | | Grade2 | | Grade3 | | Grade4 | | Grade1 | | Grade2 | | Grade3 | | Grade4 | |
| Toxicity | No. | % | No. | % | No. | % | No. | % | No. | % | No. | % | No. | % | No. | % |
| Nausea | 99 | 61.1 | 27 | 16.7 | 3 | 1.8 | 0 | 0.0 | 107 | 64.4 | 21 | 12.7 | 3 | 1.8 | 0 | 0.0 |
| Vomiting | 43 | 26.5 | 4 | 2.5 | 2 | 1.2 | 0 | 0.0 | 45 | 27.0 | 8 | 5.0 | 4 | 2.4 | 0 | 0.0 |
| Diarrhea | 33 | 20.4 | 8 | 5.0 | 3 | 1.9 | 0 | 0.0 | 42 | 25.3 | 6 | 3.6 | 2 | 1.2 | 0 | 0.0 |
| HFS | 65 | 40.1 | 14 | 8.6 | 2 | 1.2 | 0 | 0.0 | 72 | 43.3 | 12 | 7.2 | 4 | 2.4 | 0 | 0.0 |
| Anemia | 84 | 51.9 | 40 | 24.7 | 2 | 1.2 | 0 | 0.0 | 86 | 51.8 | 53 | 31.9 | 1 | 0.1 | 0 | 0.0 |
| Neutropenia | 10 | 6.1 | 56 | 34.6 | 58 | 35.8 | 0 | 0.0 | 66 | 36.7 | 76 | 45.8 | 4 | 2.4 | 0 | 0.0 |

Abbreviations: HFS, hand-foot syndrome; CT, chemotherapy; CRT, chemoradiotherapy

Table S2. Univariate and Multivariate analyses of prognostic factors for all patients for DFS.

|  | numbers | univariate | |  | multivariate | |
| --- | --- | --- | --- | --- | --- | --- |
|  |  | HR95%CI | P |  | HR95%CI | P |
| Group |  |  |  |  |  |  |
| CT | 162 | 1.000 |  |  | 1.000 |  |
| CRT | 166 | 0.745(0.565-0.983) | 0.038 |  | 0.761(0.576-1.006) | 0.056 |
| Gender |  |  |  |  |  |  |
| Male | 239 | 1.000 |  |  |  |  |
| Female | 89 | 1.187(0.878-1.606) | 0.265 |  |  |  |
| Age | 328 | 1.005(0.991-1.019) | 0.500 |  |  |  |
| Smoking history |  |  |  |  |  |  |
| No | 211 | 1.000 |  |  |  |  |
| Yes | 117 | 0.958(0.716-1.281) | 0.771 |  |  |  |
| Alcohol history |  |  |  |  |  |  |
| No | 245 | 1.000 |  |  |  |  |
| Yes | 82 | 0.936(0.680-1.288) | 0.683 |  |  |  |
| Type of gastrectomy |  |  |  |  |  |  |
| BI | 61 | 1.000 |  |  |  |  |
| BII | 180 | 1.122(0.772-1.630) | 0.546 |  |  |  |
| Roux-en-y | 87 | 0.882(0.574-1.357) | 0.568 |  |  |  |
| Tumor Differentiation |  |  |  |  |  |  |
| Poor | 283 | 1.000 |  |  |  |  |
| Medium to well | 45 | 0.794(0.518-1.216) | 0.289 |  |  |  |
| Lauren Type |  |  |  |  |  |  |
| Intestinal | 107 | 1.000 |  |  |  |  |
| Diffused | 167 | 1.160(0.847-1.588) | 0.356 |  |  |  |
| Others | 53 | 1.209(0.789-1.853) | 0.382 |  |  |  |
| N stage |  |  |  |  |  |  |
| 0 | 54 | 1.000 |  |  | 1.000 |  |
| 1 | 44 | 0.549(0.315-0.958) | 0.035 |  | 0.559(0.320-0.975) | 0.040 |
| 2 | 88 | 0.670(0.430-1.044) | 0.077 |  | 0.718(0.452-1.139) | 0.160 |
| 3 | 142 | 1.388(0.950-2.028) | 0.091 |  | 1.229(0.703-2.149) | 0.469 |
| Lymph node ratio ROC |  |  |  |  |  |  |
| 0-50% | 244 | 1.000 |  |  | 1.000 |  |
| 51%-100% | 84 | 2.023(1.506-2.718) | 0.000 |  | 1.489(1.017-2.207) | 0.041 |
| No. of lymph nodes dissected | 328 | 0.995(0.981-1.008) | 0.426 |  |  |  |
| No. of involved lymph nodes | 328 | 1.043(1.02-1.066) | 0.000 |  | 0.980(0.938-1.023) | 0.361 |
| TNM stage |  |  |  |  |  |  |
| II-IIIB | 271 | 1.000 |  |  | 1.000 |  |
| IIIC | 57 | 2.086(1.502-2.896) | 0.000 |  | 1.439(0.940-2.203) | 0.094 |
| HER2 expression |  |  |  |  |  |  |
| Negative | 291 | 1.000 |  |  |  |  |
| Positive | 22 | 1.153(0.681-1.954) | 0.596 |  |  |  |
| Unknown | 15 | 0.922(0.472-1.802) | 0.813 |  |  |  |

HR: the abbreviation of Hazard Ratio.

*CI*: the abbreviation of Confidence Interval.

TableS3. Univariate and Multivariate analyses of prognostic factors for all patients for OS.

|  | numbers | univariate | |  | multivariate | |
| --- | --- | --- | --- | --- | --- | --- |
|  |  | HR95%CI | P |  | HR95%CI | P |
| Group |  |  |  |  |  |  |
| CT | 162 | 1.000 |  |  | 1.000 |  |
| CRT | 166 | 0.756(0.570-1.003) | 0.052 |  | 0.765(0.575-1.016) | 0.064 |
| Gender |  |  |  |  |  |  |
| Male | 239 | 1.000 |  |  |  |  |
| Female | 89 | 1.186(0.869-1.606) | 0.288 |  |  |  |
| Type of gastrectomy |  |  |  |  |  |  |
| BI | 61 | 1.000 |  |  |  |  |
| BII | 180 | 1.153(0.786-1.692) | 0.467 |  |  |  |
| Roux-en-y | 87 | 0.909(0.586-1.411) | 0.671 |  |  |  |
| Tumor Differentiation |  |  |  |  |  |  |
| Poor | 283 | 1.000 |  |  |  |  |
| Medium to well | 45 | 0.845(0.551-1.296) | 0.440 |  |  |  |
| Lauren Type |  |  |  |  |  |  |
| Intestinal | 107 | 1.000 |  |  |  |  |
| Diffused | 167 | 1.122(0.884-1.682) | 0.226 |  |  |  |
| Others | 53 | 1.236(0.800-1.910) | 0.340 |  |  |  |
| N stage |  |  |  |  |  |  |
| 0 | 54 | 1.000 |  |  | 1.000 |  |
| 1 | 44 | 0.593(0.335-1.051) | 0.073 |  | 0.605(0.341-1.071) | 0.085 |
| 2 | 88 | 0.670(0.424-1.058) | 0.086 |  | 0.707(0.439-1.139) | 0.155 |
| 3 | 142 | 1.563(1.060-2.304) | 0.024 |  | 1.331(0.758-2.339) | 0.320 |
| Lymph node ratio ROC |  |  |  |  |  |  |
| 0-50% | 244 | 1.000 |  |  | 1.000 |  |
| 51%-100% | 84 | 2.131(1.580-2.874) | 0.000 |  | 1.468(0.993-2.172) | 0.055 |
| No. of lymph nodes dissected | 328 | 0.999(0.986-1.012) | 0.886 |  |  |  |
| No. of involved lymph nodes | 328 | 1.049(1.026-1.073) | 0.000 |  | 0.986(0.944-1.030) | 0.552 |
| TNM stage |  |  |  |  |  |  |
| II-IIIB | 49 | 1.000 |  |  | 1.000 |  |
| IIIC | 81 | 2.252(1.615-3.140) | 0.000 |  | 1.468(0.933-2.203) | 0.100 |
| HER2 expression |  |  |  |  |  |  |
| Negative | 291 | 1.000 |  |  |  |  |
| Positive | 22 | 1.237(0.729-2.096) | 0.431 |  |  |  |
| Unknown | 15 | 0.894(0.440-1.818) | 0.758 |  |  |  |

HR: the abbreviation of Hazard Ratio.

*CI*: the abbreviation of Confidence Interval

TableS4. Pattern of Recurrence in all patients

|  | CT  （n=162） | CRT  （n=166） | χ^2^ | *P* |
| --- | --- | --- | --- | --- |
| LRR | 32(19.8) | 20(12.0) | 3.649 | 0.063 |
| DM | 65(40.1) | 58(34.9) | 0.940 | 0.332 |
| Unknown | 19(11.7) | 24(14.5) | 0.536 | 0.464 |

Abbreviations：CT, chemotherapy；CRT，chemoradiotherapy

Locoregional recurrence：all within radiotherapy field including anastomosis site，abdominal lymph nodes No.1-16.

Distant recurrence：outside radiotherapy field including other lymph nodes，peritoneal seeding，extra-abdominal metastases.

TableS5. Pattern of Recurrence in patients with different staging

|  | II-IIIB  （n=271） | IIIC  （n=57） | χ^2^ | *P* |
| --- | --- | --- | --- | --- |
| LRR | 43(37.6) | 7(12.3) | 0.469 | 0.494 |
| DM | 87(32.1) | 36(63.2) | 19.378 | 0.000 |
| Unknown | 39(14.4) | 6(10.5) | 0.594 | 0.441 |

Abbreviations：CT, chemotherapy；CRT，chemoradiotherapy

Locoregional recurrence：all within radiotherapy field including anastomosis site，abdominal lymph nodes No.1-16.

Distant recurrence：outside radiotherapy field including other lymph nodes，peritoneal seeding，extra-abdominal metastases.

TableS6. Pattern of Recurrence in patients with different staging

|  | II-IIIB | | χ^2^ | *P* | IIIC | | χ^2^ | *P* |
| --- | --- | --- | --- | --- | --- | --- | --- | --- |
|  | CT  （n=135） | CRT  （n=136） |  |  | CT  （n=27） | CRT  （n=30） |  |  |
| LRR | 27(20.0) | 16(11.8) | 3.442 | 0.064 | 3(11.1) | 4(13.3) | 0.065 | 0.799 |
| DM | 47(34.8) | 40(29.4) | 0.907 | 0.341 | 18(66.7) | 18(60.0) | 0.271 | 0.602 |
| Unknown | 19(14.1) | 20(14.7) | 0.022 | 0.882 | 2(7.4) | 4(13.3) | 0.530 | 0.467 |

Abbreviations：CT, chemotherapy；CRT，chemoradiotherapy

Locoregional recurrence：all within radiotherapy field including anastomosis site，abdominal lymph nodes No.1-16.

Distant recurrence：outside radiotherapy field including other lymph nodes，peritoneal seeding，extra-abdominal metastases.

Table S7. Baseline of lymph node invasion between TJ-ARK01, ARTIST1 and ARTIST2

|  | TJ-ARK01 | | ARTIST1 | | ARTIST2 | | | |
| --- | --- | --- | --- | --- | --- | --- | --- | --- |
| LNs | CT | CRT | XP | XP/XRT/XP | Total | S-1 | SOX | SOXRT |
| Dissected numbers of LNs | 22  (4-69) | 21  (2-67) | 40  (13-142) | 40  (12-84) | 43  (12-101) | 42  (16-99) | 45  (12-95) | 43  (20-101) |
| Positive numbers of LNs | 6  (0-27) | 7  (0-24) | 3  (0-50) | 3  (0-51) | 5  (1-66) | 6  (1-33) | 4  (1-42) | 6  (1-66) |
| Positive LN ratio | 0.28  (0.00-1.00) | 0.35  (0.00-1.00) | - | - | 0.13  (0.01-0.91) | 0.13  (0.01-0.91) | 0.10  (0.02-0.83) | 0.15  (0.01-0.71) |
